# Tuberous sclerosis complex is a novel, amyloid-independent tauopathy associated with elevated phosphorylated 3R/4R tau aggregation

**DOI:** 10.1186/s40478-022-01330-x

**Published:** 2022-03-03

**Authors:** Andy J. Liu, Jay B. Lusk, John Ervin, James Burke, Richard O’Brien, Shih-Hsiu J. Wang

**Affiliations:** Duke University, 932 Morreene Rd, Durham, NC USA

**Keywords:** Tuberous Sclerosis Complex, Alzheimer’s disease, Phosphorylated tau, Tauopathy, 3R tauopathy, 4R tauopathy

## Abstract

Tuberous sclerosis complex (TSC) is a neurodevelopmental disorder caused by mutations in the *TSC1* and *TSC2* genes and autosomal dominantly inherited. These mutations cause hyperactivation of the mammalian Target of Rapamycin (mTOR) pathway, leading to the development of nonmalignant masses involving various organ systems. Patients with TSC also experience neuropsychiatric symptoms collectively termed Tuberous Sclerosis Complex Associated Neuropsychiatric Disorder (TAND). Due to research advancements in TSC, patients now live well beyond the age of 50. Many experience objective impairment of memory and executive function, supported by formal neuropsychological testing, beginning in their late 40s. Biomarker analysis has described elevated levels of phosphorylated tau-181 in the cerebrospinal fluid of patients with TAND. Tau-PET imaging has also shown focal accumulation of the radiotracer flortaucipir (AV1451), suggesting that TSC may be a neurodegenerative disorder arising from accumulation of phosphorylated tau. However, the flortaucipir tracer has been reported to have significant off-target binding, preventing definitive conclusions from being drawn about the molecular etiology of neurodegeneration in TSC. Therefore, we initiated the Colocalization of AV1451 and Phosphorylated Tau in Adult brain tissue (CAPA) study. This study aimed to determine if flortaucipir is bound to phosphorylated tau in brains of patients with TSC and further sought to determine the specific tau isoform seen in TSC. Our results show that flortaucipir labels the 3R/4R isoform of phosphorylated tau, commonly seen in Alzheimer’s disease. However, amyloid staining was negative in brains of adult patients with TSC. Therefore, we conclude that TAND symptoms are due to the accumulation of the phosphorylated tau isoform seen in Alzheimer’s disease. This study suggests that hyperactivation of the mammalian Target of Rapamycin pathway may play a role in the amyloid-independent development of 3R/4R tau aggregation. Our findings could lead to a new era of anti-tau therapies used to treat both disorders.

## Introduction

Various isoforms of phosphorylated tau have been described as a neuropathological substrate for several neurodegenerative diseases. This group of disorders are commonly referred to as tauopathies. The 3R and 4R isoforms of phosphorylated tau are well established [4] and are associated with various neurodegenerative conditions: the 3R tau is predominantly seen in Pick’s disease, 4R tau predominantly in progressive supranuclear palsy (PSP) and corticobasal degeneration (CBD), and a mixture of 3R and 4R (3R/4R) tau in Alzheimer’s disease (AD).

Tuberous Sclerosis Complex (TSC) is a neurodevelopmental disorder arising primarily from mutations in *TSC1* (hamartin) and *TSC2* (tuberin) (*TSC1/2)* [8]. These mutations promote the growth of benign masses involving several organ systems. In addition to the physical manifestations of TSC, these patients frequently suffer from behavioral, cognitive and psychiatric difficulties collectively termed Tuberous Sclerosis Complex Associated Neuropsychiatric disorders (TAND) [14]. Our work published in 2019 suggested a significant overlap between TSC and a group of neurodegenerative disorders named Frontotemporal Dementias (FTD) based on neuropsychological testing, however, TSC subjects scored significantly worse on memory when compared to FTD subjects [5]. Cerebrospinal fluid (CSF) results showed elevated phosphorylated tau (pTau-181) levels with an average of 32 pg/mL in TSC subjects when compared to healthy controls [5]. Furthermore, AV1451 (Flortaucipir)-PET/CT scans were completed in 3 adult patients with TSC. Intriguingly, focal uptake of AV1451in the frontal and temporal regions was seen in all 3 of these patients. Collectively, based on the significant memory loss and elevated CSF pTau-181 levels, it was hypothesized that TSC may represent a novel genetic tauopathy similar to that seen in AD.

AV1451 has a high affinity for pTau-181 related to Alzheimer’s disease by PET imaging [6]; however, AV1451 also displays significant off-target binding [1]. As a result, we could not definitively conclude that the focal binding of AV1451 in adult patients with TSC was due to the accumulation of pTau-181. Our current study investigates whether the AV1451 signal corresponds to tau pathology in adult patients with TSC by comparing AV1451 staining and immunohistochemical staining of various tau antibodies on brain tissue from adult patients with TSC, AD, FTLD-tau, and healthy controls. This is based on prior studies which have shown that TSC is an infantile tauopathy accumulating phosphorylated tau [10]. The isoform of phosphorylated tau in infantile tauopathies is still an area of active investigation. Here we show that AV1451 labels sparse neurofibrillary tangles in the entorhinal cortex, dorsolateral prefrontal cortex (Brodmann area 8) and inferior frontal cortex (Brodmann area 11) of adult patients with TSC. The tangles are also labeled by AT8 (which detects pTau-202/205) and GT-38 (which detects a conformation-specific mixture of 3R- and 4R-tau). Amyloid plaques were not detected in any of the brains from adult patients with TSC. These results suggest that TSC is a genetic 3R/4R tauopathy that develops independent of amyloid plaques seen in AD.

## Materials and methods

### Antibodies and PET ligand

The antibodies used for this study were AT8 (ThermoScientific cat # MN1020), GT-38 (ABCAM cat # ab254274), and 4G8 (BioLegend Cat#: 800,702). Unlabeled AV1451 was generously provided by Avid Radiopharmaceuticals.

### Brain tissue

Tissue from cognitively healthy controls (HC), and from patients with AD, FTLD-tau, and TSC were obtained from the NIH biobank. Brain tissue was age and gender matched to HCs. AD brain tissue was requested with Braak stage 4 and above. Three subjects carried a clinical diagnosis of Down Syndrome (DS) and were included in the AD cohort. FTD brain tissue demonstrating FTLD-tau pathology was requested. Since patients with TAND showed executive function, behavioral and memory difficulties, three brain areas involving these functions, the dorsolateral prefrontal cortex (Brodmann area 8), inferior Frontal Cortex (Brodmann area 11) and the entorhinal cortex were selected for neuropathological analysis. Formalin fixed paraffin embedded (FFPE) tissue was sectioned at eight microns on a Leica microtome.

### Immunohistochemistry (IHC)

IHC was performed on formalin fixed paraffin embedded (FFPE) sections that were deparaffinized in xylene and washed in absolute ETOH and 95% ETOH. Endogenous peroxidase activity was blocked with 1.875% H_2_O_2_ in methanol for 8 min. Samples were then washed and hydrated in DH_2_O. GT38 (ABCAM: cat#ab254274) required antigen retrieval with a boiling citrate buffer at pH6 for 30 min. A second blocking step was applied by submerging the sections in a dish containing 200 mL of 5% w/v nonfat dry milk in 0.05 M Tris buffer, pH7.6, for 20 min at room temperature. Primary antibodies were applied to each slide (1:500 for AT8 and GT-38) and allowed to incubate for 45 min at 37–40°C. Secondary antibodies were applied using the Dako EnVision™ Dual Link System-HRP (Agilent Technologies, Cat# K406189-2) and incubated for 30 min at 37–40°C. The slides were then developed with Dako DAB solution (Agilent Technologies, Cat# K346811-2) for 5 min and counterstained with Fisherfinest Hematoxylin + (ThermoFisher, Cat#220-100) for 15–25 sec. The slides were dehydrated through graded alcohols, cleared in xylene, and coverslipped in Permount mounting medium.

### AV1451 staining

The AV1451 PET Ligand is autofluorescent and was applied to FFPE sections. The sections were first deparaffinized in xylene and washed with absolute ETOH, 95% and 70% ETOH. Endogenous autofluorescence was blocked using 0.1% Sudan Black B solution made with 70% ETOH. The AV1451 PET Ligand was supplied as a powder in a 1 mg vial. Then 380 µL of DMSO was added to the powder and allowed to dissolve to make a 10 mM stock solution. The working solution of AV1451 was a 1:100 dilution applied for 1 h at room temp. The sections were rinsed in 50% ETOH for 10 min prior to rinsing in DH_2_O for 10 min. The sections were coverslipped using Vectashield Hardset(Vector Laboratories; Cat#H1400).

### Imaging and data analysis

AV1451 signal was analyzed using a digital Revolve R3 fluorescent microscope (ECHO, San Diego, CA) using the DAPI channel. AT8 and GT-38 immunohistochemistry was analyzed using an Olympus bright field microscope. Five 1mm^2^ fields with the highest number of tangles were chosen from each slide. The average number of tangles was quantified and converted to a semi-quantitative score as follows, 0: no tangles, 1: < 1 tangle per 1mm^2^ field, 2: 1–2 tangles per 1mm^2^ field, 3: 3–5 tangles per 1mm^2^ field, 4: 6–10 tangles per 1mm^2^ field, and 5: > 10 tangles per 1mm^2^ field.

## Results

### Patient characteristics

Patient characteristics are summarized in Table 1. Thirty-five patients were included in the study, including 11 healthy control patients, 9 patients with AD, 10 patients with TSC, and 5 patients neuropathologically diagnosed with FTLD-tau. Notably, no HC patients or patients with FTLD-tau had epilepsy, whereas 50% of patients with TSC had epilepsy and 22% of patients with AD had epilepsy. Similarly, no control patients, patients with AD, or patients with FTD had intellectual or developmental disability (IDD), but 55% of patients with TSC had Intellectual or Developmental Disability (IDD).

**Table 1:** Demographic and clinical characteristics of the study cohort by diagnosis

	Control (N = 11)	DS/AD (N = 9)^a^	TSC (N = 10)	FTD (N = 5)^b^
Age (Mean, SD)	52 (9)	53 (6)	49 (6)	69 (4)
Race (N, percent)				
Black	1 (9%)	1 (11%)	0 (0%)	0 (0%)
White	8 (73%)	8 (89%)	10 (100%)	4 (80%)
Unable to determine	2 (18%)	0 (0%)	0 (0%)	1 (20%)
Sex (percent female)	46%	56%	50%	80%
Epilepsy diagnosis (N, Percent)				
Epilepsy present	0 (0%)	2 (22%)	5 (50%)	0 (0%)
Epilepsy absent	11 (100%)	6 (67%)	3 (30%)	5 (100%)
Unable to Determine	0 (0%)	1 (11%)	2 (20%)	0 (0%)
IDD diagnosis (N, Percent)	0 (0%)			
IDD Present	0 (0%)	0 (0%)	5 (55%)	0 (0%)
IDD Absent	11 (100%)	8 (89%)	3 (33%)	5 (100%)
Unable to determine	0 (0%)	1 (11%)	2 (22%)	0 (0%)

*IDD* Intellectual or developmental disability

^a^Three subjects carried a clinical diagnosis of Down Syndrome

^b^Four subjects had progressive supranuclear palsy (PSP), one had FTLD-tau, unclassified

### Qualitative immunohistochemistry findings

**Fig. 1:**
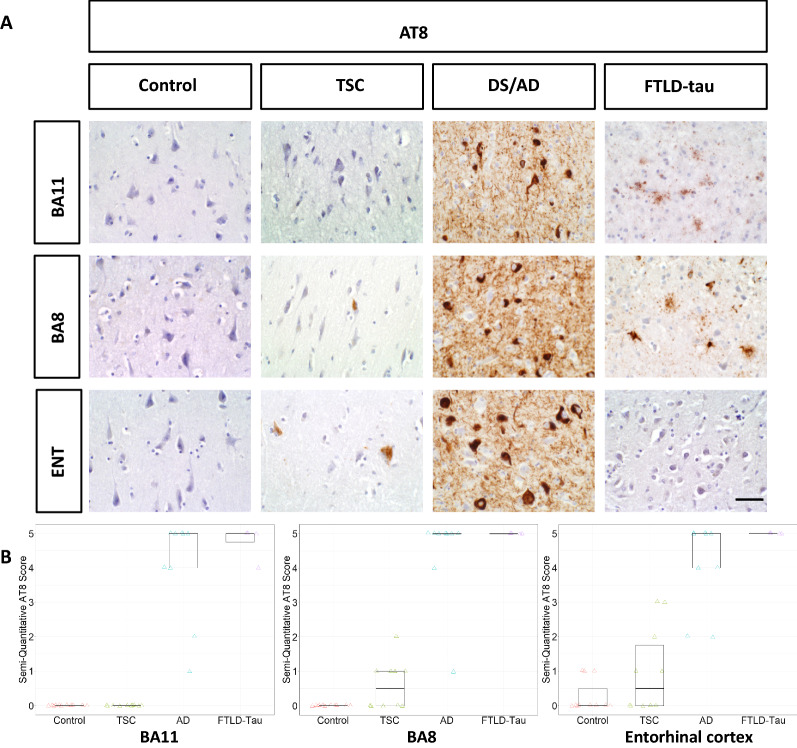
**a** Representative images of BA11, BA8, and entorhinal cortex from control, TSC, DS/AD, and FTLD-tau patients immunostained with AT8. Scale bar = 50 µm. **b** Semi-quantitative analysis is represented in box plots

**Fig. 2:**
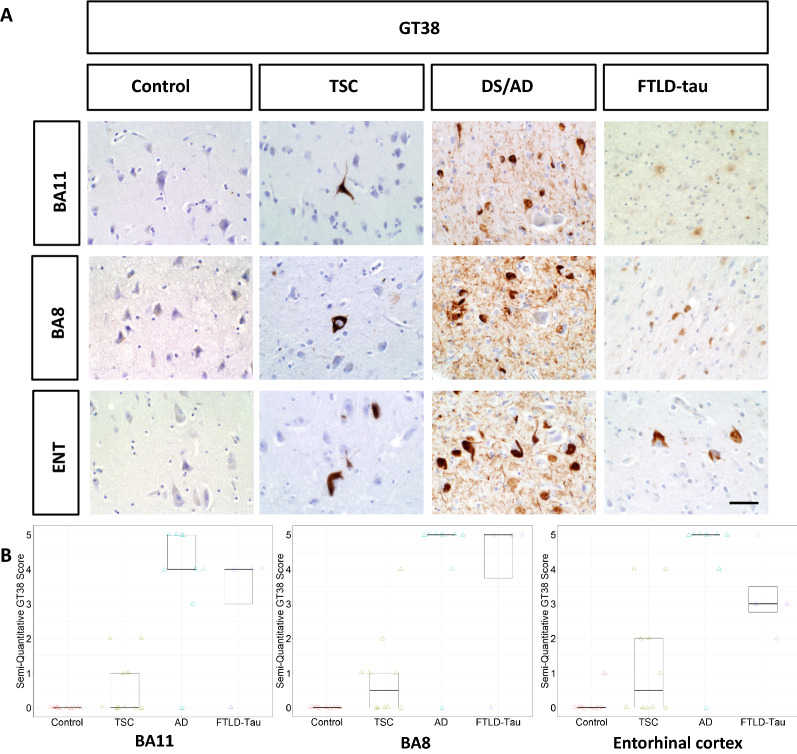
**a** Representative images of BA11, BA8, and entorhinal cortex from control, TSC, DS/AD, and FTLD-tau patients immunostained with GT38. Scale bar = 50 µm. **b** Semi-quantitative analysis is represented in box plots

**Fig. 3:**
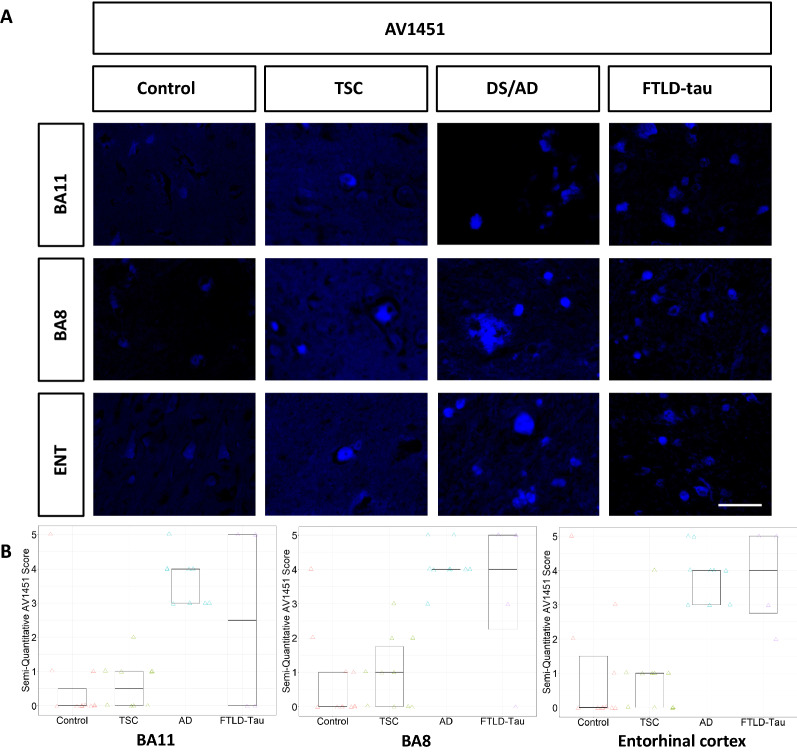
**a** Representative images of BA11, BA8, and entorhinal cortex from control, TSC, DS/AD, and FTLD-tau patients immunostained with AV1451. Scale bar = 50 µm. **b** Semi-quantitative analysis is represented in box plots

**Fig. 4:**
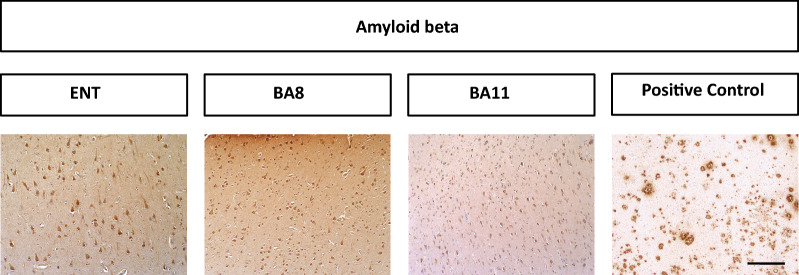
Representative images of BA11, BA8, and entorhinal cortex from TSC patients immunostained with 4G8. No amyloid plaques were observed

Patients with TSC exhibited sparsely positive AT8 staining in the entorhinal cortex and BA8 but not BA11. AT8 staining in patients with TSC was considerably sparser than in patients with DS/AD patients or FTLD-tau. HC patients did not show appreciable AT8 staining in any region (Fig. 1). Patients with TSC exhibited more strongly positive GT38 (3R tau + 4R tau) staining in all three brain regions studied, although the density was still sparse compared to patients with DS/AD or FTLD-tau (Fig. 2). AV1451 staining was positive for neurofibrillary tangles in patients with TSC, FTLD-tau, and DS/AD. AV1451 also labeled neuritic plaques from patients with DS/AD, but no glial tau lesions were observed (Fig. 3). No patients with TSC exhibited glial-tau lesions such as coiled bodies, tufted astrocytes, or astrocytic plaques (Figs. 1, 2, 3). Furthermore, no patients with TSC had amyloid-beta plaques (Fig. 4). The percentage of patients from each cohort positive for AT8, GT38, and AV1451 in the studied brain regions is sunmmarized in Table 2.

**Table 2:** Percentage of patients from each cohort positive for AT8, GT38, and AV1451 in the sampled brain regions

		Control N = 11	DS/AD N = 9	TSC N = 10	FTLD-tau N = 5
AT8	Entorhinal Cx	3 (27%)	9 (100%)	5 (50%)	5 (100%)
	BA8	0	9 (100%)	5 (50%)	5 (100%)
	BA11	0	9 (100%)	0	4 (80%)
GT38	Entorhinal Cx	1 (9%)	8 (90%)	5 (50%)	4 (80%)
	BA8	0	8 (90%)	5 (50%)	4 (80%)
	BA11	0	8 (90%)	4 (40%)	4 (80%)
AV1451	Entorhinal Cx	3 (27%)	9 (100%)	6 (60%)	5 (100%)
	BA8	3 (27%)	9 (100%)	6 (60%)	4 (80%)
	BA11	2 (18%)	9 (100%)	5 (50%)	3 (60%)

### Semi-quantitative immunohistochemistry quantification

Semi-quantitative analysis was utilized to provide more formal assessment of qualitative findings. For AT8 (Fig. 1B), we observed that control patients and patients with TSC had no AT8 staining in BA11, whereas the median scores for patients with DS/AD and FTLD respectively were 5 (> 10 tangles/1 mm^2^ field). In BA8, however, patients with TSC had a median score of 0.5 (< 1 tangle/1 mm^2^ field, but tangles present), compared to controls which lacked AT8 staining. The median AT8 score for patients with TSC was also 0.5 in the entorhinal cortex, but the 75th percentile score was 1.75, as more patients had AT8 scores of 2 (1–2 tangles/1 mm^2^ field) or 3 (3–5 tangles/1 mm^2^ field). Median AT8 scores were high in all three brain regions for patients with DS/AD and FTLD-tau.

For GT38 scoring (Fig. 2B), while the median GT38 score in BA11 for patients with TSC was 0, the 75th percentile score was 1 (< 1 tangle/1 mm^2^ field), as several patients had scores of 1 or 2 (1–2 tangles/1mm^2^ field). The median GT38 score in BA8 was 0.5, and the 75th percentile score was 1; the median GT38 score in entorhinal cortex was 0.5, whitle the 75th percentile score was 2, as some patients had scores up to 4 (6–10 tangles /1mm^2^ field). Patients with AD and FTLD had high median GT38 scores in all three brain regions. Control patients had minimal to no appreciable GT38 scoring in all three brain regions.

Finally, AV1451 scores (Fig. 3B) also varied across groups. Patients with TSC had median scores of 0.5, 1, and 1 in BA11, BA8, and the entorhinal cortex respectively, compared to control patients with a median score of 0 in all regions. Patients with AD and FTLD-tau had median AV1451 scores greater than 2 in all three brain regions.

## Discussion

The etiology of TAND behaviors remains unknown and has been hypothesized to be related to seizures, structural changes, and intellectual disability [12–14]. Our study suggests an additional neuropathological etiology. Future studies are needed to elucidate if the neuropathological phenotypes correlate with previously described neuroradiologic phenotypes and clinically meaningful TAND clusters [11, 13].

Our study provides the first compelling evidence that TAND is a novel amyloid-independent 3R/4R tauopathy. Our finding that patients with TSC had elevated density of AT8 staining, which identifies tau phosphorylated at Ser202 and/or Thr205, compared to control patients, but less than patients with AD or FTLD suggests that phosphorylated tau accumulates to some extent in patients with TSC. Additionally, our finding that the density of GT38 positive tangles was higher in patients with TSC than control patients, and again lower than patients with AD/FTLD, suggests that TSC is a mixed 3R/4R tauopathy. Patients with TSC notably lacked amyloid plaques and glial-tau lesions, distinguishing the pathology underlying TAND from other neurodegenerative conditions.

It is not entirely clear why patients with TAND had a lower density of GT38 positive tangles than FTLD patients; one possible explanation is that TSC causes a diminished extent of pathological 3R/4R tau accumulation compared with classic FTLD due to a different underlying causative mechanism. Prior research has suggested that *TSC1/2* mutations, excessive mTOR signaling, and dementias may be linked mechanistically [9]. A recent case–control study likewise suggested that pTau-181 was elevated in patients with TAND and that these patients had symptoms consistent with a neurodegenerative condition [5]. Our study extends this body of literature by describing the precise underlying neuropathological phenotype of patients with TAND. In particular, the hyperactivation of mTOR signaling seen in TSC could contribute to increased tau protein aggregation and the development of 3R/4R positive tangles. This finding, if true, would have broad implications for research into other neurodegenerative conditions, as it would imply that mTOR signaling pathway could be causally related to the formation of neurofibrillary tangles, but not neuritic plaques [3, 7]. Additional characterization between pTau distribution (and density) and clinical phenotypes has major implications both for the diagnosis and management of TAND in patients with TSC and for the pathogenesis of 3R/4R tau commonly seen in Alzheimer’s disease.

Another amyloid independent 3R/4R tauopathy named Primary Age Related Tauopathy (PART) was considered to explain the findings in the TSC cohort. What makes PART less likely is the age at which the pTau was found. The mean age of the TSC cohort was 49 (Table 1). In an autopsy study, 20–25% of patients age 90 or older were found to have PART [2]. More importantly, PART is defined by the accumulation of 3R/4R pTau in the mesial temporal lobes. The TSC cohort from this study displayed 3R/4R pTau in both the frontal and temporal lobes. For these reasons, PART is unlikely to explain our findings in the TSC cohort. Future research is needed to determine the precise mechanism through which mTOR signaling pathway may be related to the development of neurofibrillary tangles.

Strengths of this study include its comparison to age and gender matched healthy controls, as well as its simultaneous evaluation of multiple neuropathologies, including AD, TSC, FTLD-tau, and other neurodegenerative disorders. Additional strengths include detailed, semi-quantitative neuropathological evaluation of various tau isoforms, allowing for precise definition of the specific tauopathy seen in patients with TAND.

This study has limitations. First and foremost, its sample size was small, as TAND is a rare condition, which prevented us from utilizing formal tests of statistical significance for the associations observed. Additionally, this study was unable to control for intellectual ability/cognitive status, which has been suggested to be a major risk factor for the clinical presentation of TAND [12]. Despite these limitations, this study suggests that TAND is a novel 3R/4R tauopathy independent of amyloid plaque formation, and points to promising new directions for research into the mechanism of TAND symptoms and novel anti-tau therapies in AD.

## Data Availability

Data sharing not applicable to this article as no datasets were generated or analysed during the current study.
